# Proposal of a novel APPLE-KNOT undulator by selecting the KNOT field as the dominant magnetic field

**DOI:** 10.1107/S1600577526000159

**Published:** 2026-01-30

**Authors:** Binghao Zhang, Chao Chen, Yuanfang Xu, Zhouyu Zhao, Heting Li

**Affiliations:** a National Synchrotron Radiation Laboratory University of Science and Technology of China Hefei230029 People’s Republic of China; SESAME, Jordan

**Keywords:** APPLE-KNOT undulator, period ratio, field strength ratio

## Abstract

The APPLE-KNOT undulator forms composite magnetic fields by superimposing an APPLE field and a KNOT field with different period lengths, in which one serves as the dominant field to approach the target photon energy and the other acts as an additional component to transversely deflect the electron beam away from the axis. Variable polarization modes can be realized with a low on-axis heat load in the APPLE-KNOT undulator. In previous studies, APPLE arrays with shorter period length have a stronger field strength than KNOT arrays with longer period length. However, a sharp reduction in flux and an obvious degeneration in polarization degree of circular polarization mode can be observed. In this paper, such a phenomenon is theoretically studied and explained in detail. A novel undulator in which the KNOT array configuration, conventionally used for the weak field, is instead utilized to generate a strong field. Different from the traditional APPLE-KNOT undulator, the KNOT and APPLE magnet blocks are correspondingly merged to form two new blocks with significantly different specifications. The simulation results indicate that both the flux and polarization degree of circular polarization mode can be effectively improved, which are highly consistent with the theoretical prediction. The merged magnet arrays keep a symmetric structure within the single undulator period that always ensure an inherently good performance on the field integral.

## Abstract 2

The degradation of circular polarization state in conventional APPLE-KNOT undulators has been noted. To overcome this limitation, we propose a novel APPLE-KNOT undulator design which utilizes the KNOT magnetic field as the dominant component.

## 1. Introduction

Undulator radiation, characterized by high brilliance, narrow bandwidth and tunable wavelength, has been widely adopted in synchrotron radiation. Various types of undulators have been developed to meet different experimental requirements. The APPLE-type undulator is well known as the light source for tuning variable polarization mode (Sasaki *et al.*, 1993 ▸; Sasaki, 1994 ▸). The most conventional polarization-tunable undulator is the APPLE-II undulator. In circular/elliptical polarization mode, the horizontal and vertical magnetic fields have a constant phase shift and the electron velocity is always scattered away from the undulator axis, hence little heat load can be found on-axis. In contrast, for linearly polarized radiation, the transverse velocity of the electron is zero when the magnetic field reaches maximum, and then the on-axis power density reaches a peak, which could possibly become a serious heat-load problem especially for generating low-energy photons in high-energy storage rings.

**Figure 1:**
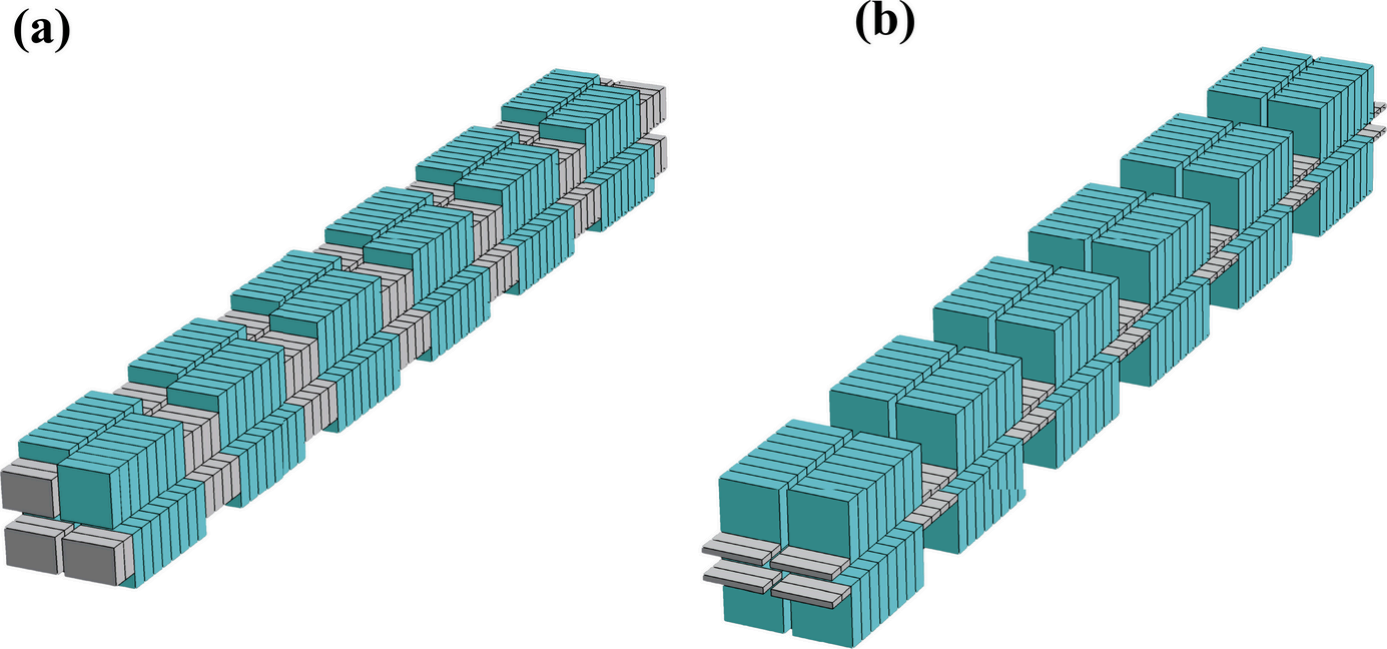
Magnetic structures of the traditional (*a*) and proposed (*b*) four-array AK undulator.

Extensive research has been done to address the issue of on-axis heat load, promoting innovative solutions over the past two decades (Sasaki *et al.*, 1998 ▸; Qiao *et al.*, 2009 ▸; Yan & Qiao, 2010 ▸; Yamamoto *et al.*, 2019 ▸; Zhao & Jia, 2022 ▸). The typical figure-8 undulator, which consists of three magnet arrays at both the top and bottom, with the period ratio of the horizontal field to the vertical field being set to 1:2, is developed to generate linearly polarized radiation with low on-axis heat load, but is not able to generate circularly polarized radiation (Tanaka & Kitamura, 1995 ▸; Tanaka & Kitamura, 1996 ▸; Tanaka *et al.*, 1999 ▸). Based on the APPLE-II undulator, the first-generation APPLE-KNOT (AK) undulator, which consists of two APPLE-II magnet arrays with a period ratio of 2:3 and eight magnet arrays, generates linearly/circularly polarized radiation while keeping a low on-axis heat load (Sasaki *et al.*, 2013 ▸). The inner four APPLE arrays are used to generate a dominant field and the outer four KNOT arrays are used to generate an additional weak field. Such an AK undulator has been successfully adopted at SSRF (Zhou *et al.*, 2016 ▸). However, its complex mechanical structure and phase adjustment, coupled with weak field strength in vertical polarization mode, result in a relatively poor performance for on-axis heat-load suppression. The second version of AK undulator was further developed by merging APPLE and KNOT magnet arrays (Ji *et al.*, 2015 ▸). The total number of magnet arrays is reduced from eight to four with a tilt magnetization angle, as shown in Fig. 1 ▸(*a*), which was firstly adopted at HEPS (Yang *et al.*, 2021 ▸; Yang *et al.*, 2022 ▸; Huang *et al.*, 2023 ▸).

The linear polarization mode can maintain a high flux and a high polarization degree; however, in circular polarization mode the flux is greatly suppressed with a relatively low polarization degree. Such a phenomenon can also be observed in other relevant studies (Ramezani Moghaddam & Cacho, 2022 ▸; Ramezani Moghaddam *et al.*, 2023 ▸).

In this paper, we propose a novel magnet-merging four-array AK undulator. Note that, here, the dominant field and additional field are only distinguished by the field strength, and are not limited by the APPLE or KNOT arrays. In the new configuration, the KNOT array is used to generate the dominant field, which is different from the traditional AK undulator whose dominant field comes from the APPLE array.

The APPLE array and KNOT array swap their original magnetic field directions in comparison with the traditional AK undulator. After magnet merging, as shown in Fig. 1 ▸(*b*), the KNOT and APPLE magnet blocks are correspondingly merged to form two new blocks with significantly different specifications. The magnet block of large size is the merged block which has tilt magnetization, while the magnet block of small size is only inherited from the APPLE array. The tilt magnetization angle of the merged magnet is limited to a small range. Both the theoretical and numerical results prove that the flux and polarization degree of circular polarization mode can be effectively improved in comparison with the previous AK undulator. In addition, the APPLE and KNOT arrays in a single period are arranged by bilateral symmetry. The termination magnet blocks keep symmetry in each period and do not have tilt magnetization, thus the first field integral is naturally small, which will be beneficial for the practical field integral compensation. The rest of the paper is organized as follows. From a theoretical perspective, Section 2 discusses the general characteristics of the circularly polarized radiation of the AK undulator, then reveals the essence of why the previous AK undulator cannot obtain a good radiation performance in circular polarization mode as well as in linear polarization mode. Inspired by the theoretical results, a novel AK undulator is proposed by selecting the KNOT field as the dominant field. The physical design of such an undulator is illustrated in Section 3, and the corresponding radiation performance compared with the traditional AK undulator is demonstrated in Section 4. Finally, Section 5 gives a discussion and summary.

## 2. Theory

In AK type undulators, various magnetic fields can be generated by superimposing the dominant field and additional field with different period lengths. In theory, the composite magnetic field of an AK undulator in circular polarization mode can be simplified as 
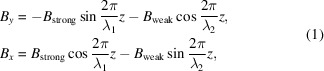
where *B*_strong_ and *B*_weak_ are the peak fields corresponding to the period lengths of λ_1_ and λ_2_, respectively. With *B*_strong_ = α*B*_weak_ = α*B*_0_, and λ_1_ = *m*λ_2_ = *m*λ_0_ (α > 1, *m* > 0, and *m* ≠ 1), we have 
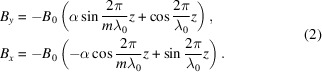
Based on the magnetic fields, the electron transverse velocity and orbit can be easily deduced. The far-field undulator radiation can be calculated as (Kim, 1986 ▸; Walker, 1993 ▸; Walker, 1998 ▸) 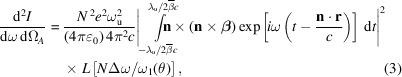
where ω_u_ is the resonance frequency of undulator and ω_1_ is the fundamental radiation frequency, and λ_u_ is the period of the AK undulator. 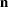 = 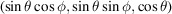 is the unit vector from the emission point to the observer. 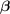 is the vector of relative velocity, and 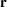 is the vector from the electron position to the observation position. *N* is the number of undulator periods and *L* is the line-shape function.

For the new proposed AK undulator, the KNOT field is stronger than the APPLE field, and therefore we have *m* = 3/2 and λ_u_ = 3λ_0_. The first items in equation (2) come from KNOT arrays. Equation (3) can be expressed as 
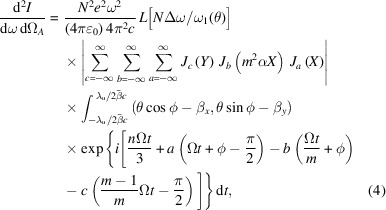
with Ω = 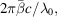
*A* = 1 + (*m*^2^α^2^ + 1)*K*^2^ + γ^2^θ^2^, *X* = 2*n*γθ*K*/3*A* and *Y* = 2*nK*^2^*m*^2^α/[3*A*(*m* − 1)].

In contrast, in the traditional AK undulator *m* is set to 2/3. The first items in equation (2) comes from the APPLE arrays. Equation (3) can be expressed as 
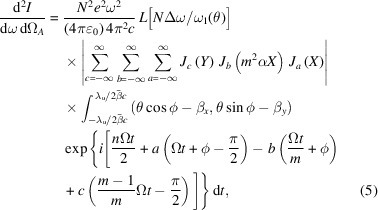
with *X* = *n*γθ*K*/*A* and *Y* = *nK*^2^*m*^2^α/[*A*(1 − *m*)].

**Figure 2:**
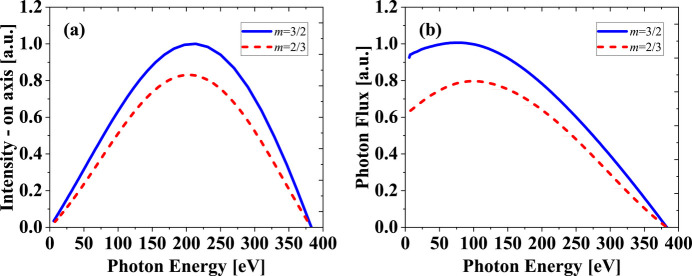
Normalized photon flux intensity (*a*) and flux (*b*) at different resonance photon energy for *n* = 2, *m* = 3/2 (solid blue) and *m* = 2/3 (dashed red), with λ_u_ = 240 mm, α = 1.5 and an electron beam energy of 2.2 GeV. The acceptance angle at different photon energy always keeps two multiples of divergence.

To make a direct comparison, we perform numerical calculations based on the two equations above with the same fundamental period and field strength ratio. The electron parameters are those of the Hefei Advanced Light Facility (HALF), a fourth-generation synchrotron radiation light source under construction in China (Zhao *et al.*, 2023 ▸). Table 1 ▸ lists the design parameters of the electron beam in the middle of a long straight section of HALF. The photon energy is scanned by changing the field strength. As shown in Fig. 2 ▸, the flux of *m* = 3/2 is higher than that of *m* = 2/3. The difference in flux density reaches maximum at an energy of 200 eV, while the flux within two multiples of divergence reaches maximum around an energy of 100 eV. The differences in photon flux between these two period ratios decreases gradually when the photon energy is above 200 eV. Furthermore, we also investigate the radiation performance with different α. With an increase of α, the differences in both flux density and total flux within two multiples of divergence are obviously increased. The calculated results at the resonance photon energy are highly consistent with results obtained using *SPECTRA* (Tanaka & Kitamura, 2001 ▸).

**Figure 3:**
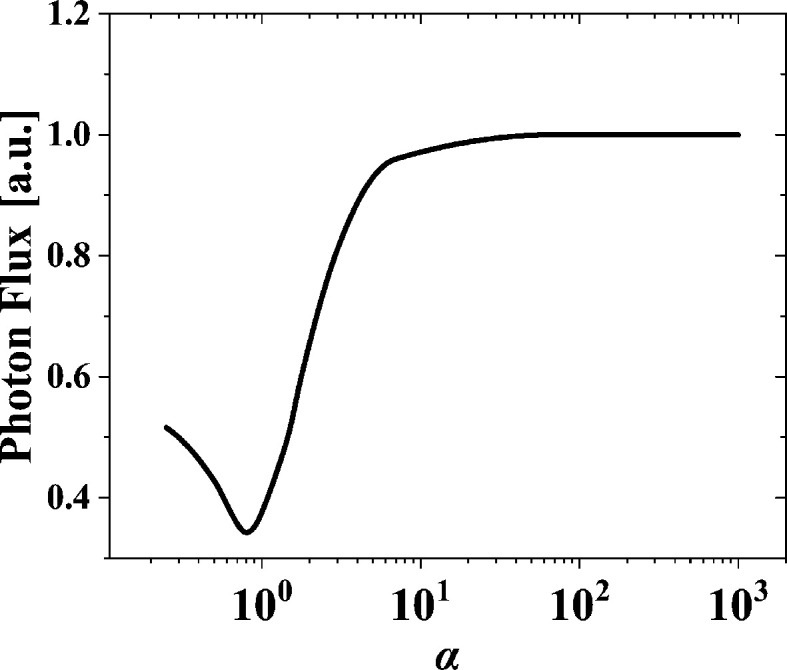
The normalized photon flux depends on the field strength ratio α with *m* = 3/2, a photon energy of 6 eV and electron beam energy of 2.2 GeV. The acceptance angle for different α always keeps two multiples of divergence.

In the AK undulator, the magnetic contributions from the APPLE and KNOT arrays are determined by the size of the magnet block and the tilt angle of magnetization. Before making a specific undulator design, here we only focus on varying the field strength ratio in theoretical models, without optimizing the magnet block specifications. Fig. 3 ▸ shows the photon flux depending on field strength ratio at a photon energy of 6 eV. The flux appears to be significantly improved with increasing α.

When the field strength ratio is more than ten, the AK undulator can almost exclude the effect of the additional field and transform into an APPLE-II undulator. Obviously, in practice, the field strength ratio cannot be set to such a high level. The field strength of the additional field must be able to effectively deflect the electron beam away from the axis to suppress the on-axis heat load.

In this paper, by choosing the KNOT field as the dominant field, the period ratio of the strong field to the weak field is set to 3:2. With a large size of the KNOT magnet block, a high field strength ratio between the KNOT and APPLE arrays can be achieved. Taking into account several factors on the field improvement above and benefiting from the period ratio 3:2 between the strong field and weak field, a significant flux improvement on circular polarization mode can be expected in the novel AK undulator.

## 3. Undulator design

Before starting the physical design of the AK undulator, an appropriate magnet merging strategy must be determined. Generally, there are two methods to merge magnet blocks. The first method directly merges APPLE and KNOT blocks with different period lengths. The merged magnet block is divided into a large number of thin magnet pieces, resulting in complex magnetization directions and complicated assembly. The termination magnet block requires additional structure design to eliminate the field integral. The second method maintains the uniform longitudinal dimension for the APPLE and KNOT blocks, and selectively removes a portion of the KNOT blocks to achieve the desired period length difference.

Since these arrays in a single period can be arranged by bilateral symmetry, the symmetry termination can be preserved after magnet merging. The first field integral is naturally small, which will be beneficial for the practical field integral compensation. Therefore, the second method with selected KNOT blocks being removed is preferred in this paper. During the magnet merging process of the novel AK undulator, the size of the KNOT block should be enlarged to reinforce the KNOT field contribution, while the APPLE field remains comparatively weak. Just as we theoretically studied in the section above, it will be beneficial for improving the radiation performance of circular polarization mode.

**Figure 4:**
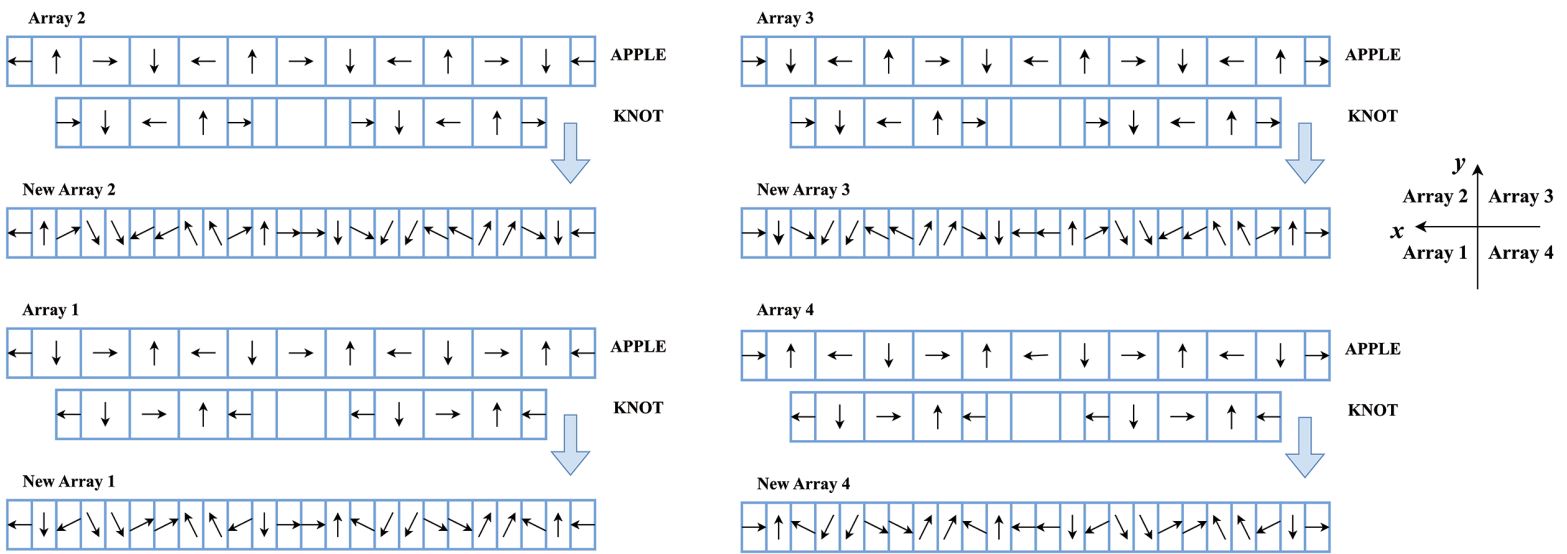
Schematic diagram of magnet merging of APPLE and KNOT arrays for the novel AK undulator. The arrow in each magnet block represents the magnetization direction, which is calculated from the field strength ratio of the APPLE to KNOT components.

A schematic diagram of the magnet merging process is shown in Fig. 4 ▸. The magnet block of the APPLE array has an identical longitudinal length as the KNOT array. Within one complete undulator period before merging the blocks, a portion of the KNOT blocks is removed, generating a period length ratio of 2:3 between APPLE (weak) field and KNOT (strong) field. For the zero phase shift case shown in Fig. 4 ▸, KNOT arrays are used to generate a vertical field and APPLE arrays are used to generate a horizontal field, which is in contrast to the traditional AK undulator. To make sure the field strength of the KNOT array is much stronger than that for the APPLE array, their size specifications should have a substantial difference before merging the blocks. The magnetization directions of the merged blocks are directly inherited from their original configurations before merging. The field strength ratio of the strong field to the weak field is adjustable, achieved by modifying a block’s dimension and magnetization direction.

In our design, after the merging process, the size ratio of the merged block to the unmerged block in the vertical direction reaches 10:1. The horizontal and longitudinal sizes of the merged and unmerged blocks remain the same. Obviously, the tilt magnetization angle cannot be too large, otherwise the field contribution from the APPLE array will be greatly improved. The longitudinal dimensions of the end magnetic blocks are half those of the remaining blocks to match the magnetic field at termination. The undulator consists of 96 blocks per complete period after magnet merging, with the longitudinal length setting to the half of the magnet block before magnet merging. In each undulator period, the magnetization directions of the first and last magnetic blocks are the same, oriented horizontally without inclination. The magnetization directions of the merged magnet arrays are pairwise opposite, leading to the natural cancellation of the first field integral.

The detailed parameters of the novel AK undulator are listed in Table 2 ▸. In our undulator design, the magnet material is NdFeB with a typical remanent magnetization of 1.26 T. The period ratio of the strong (KNOT) field to weak (APPLE) field is set to 3:2, with the field strength ratio set to 5:1. The magnetization angle of each magnet block can be determined by this specific field strength ratio. Taking the magnet array 2 for example, as shown in Fig. 4 ▸, the magnetization angles for the merged magnet blocks along the beam direction are: 180.0°, 90.0°, 12.6°, 282.6°, 282.6°, 192.6°, 192.6°, 102.6°, 102.6°, 12.6°, 90°, 0°, 0°, 270.0°, 347.4°, 257.4°, 257.4°, 167.4°, 167.4°, 77.4°, 77.4°, 347.4°, 270° and 180°, where the angles are defined counter-clockwise from the beam direction (left side to right side in each array). The magnetization vectors for all the magnet blocks in other arrays are determined consistently by this same method. To have a direct comparison between different versions of AK undulators, we also make a specific model of the traditional AK undulator, as listed in Table 3 ▸. It is partially referred to as the HEPS type AK undulator (Yang *et al.*, 2021 ▸; Yang *et al.*, 2022 ▸; Huang *et al.*, 2023 ▸). The period ratio of the strong (APPLE) field to the weak (KNOT) field is set to 2:3. For the traditional AK undulator which specifies a field strength ratio of 20:13, the magnetization angles for its array 2 shown in Fig. 1(*b*) of Yang *et al.* (2021 ▸) are: 180°, 90°, 53.3°, 323.3°, 323.3°, 233.3°, 233.3°, 143.3°, 143.3°, 53.3°, 90°, 0°, 0°, 306.7°, 216.7°, 216.7°, 216.7°, 126.7°, 126.7°, 36.7°, 36.7°, 306.7°, 270° and 180°, with the angle definition being the same as for the novel AK design.

The horizontal and vertical sizes of the merged block are the same in these two undulators. In the traditional AK undulator, the difference in field strength between APPLE and KNOT fields is not too large, therefore the size of the unmerged block must be larger than that of the novel AK undulator. The height and length of the blocks corresponding to blank segments of the novel AK and traditional AK undulators are set to 5 mm × 14 mm and 35 mm × 15 mm, respectively. The block lengths are set with a small difference to have the same minimum resonant photon energy. Normally, the AK undulator is used to reach a low photon energy and pursues a large length to satisfy the flux demand for experiment users; therefore it will inevitably introduce a large transverse size of the power distribution. In our cases, the minimum photon energy is set to 6 eV, which is determined by experiment users of HALF (Zhao *et al.*, 2023 ▸). The undulator length is set to about 4.4 m. The minimum undulator gap is set to a large value (17 mm) to avoid deflecting too much radiation power on the vacuum pipe. In addition, considering reduction of the well known negative impact on the electron beam especially for vertical polarization mode, here the clearance between the arrays is increased to 6.5 mm, which is slightly larger than that for the normal APPLE-II undulator (1–2 mm).

**Figure 5:**
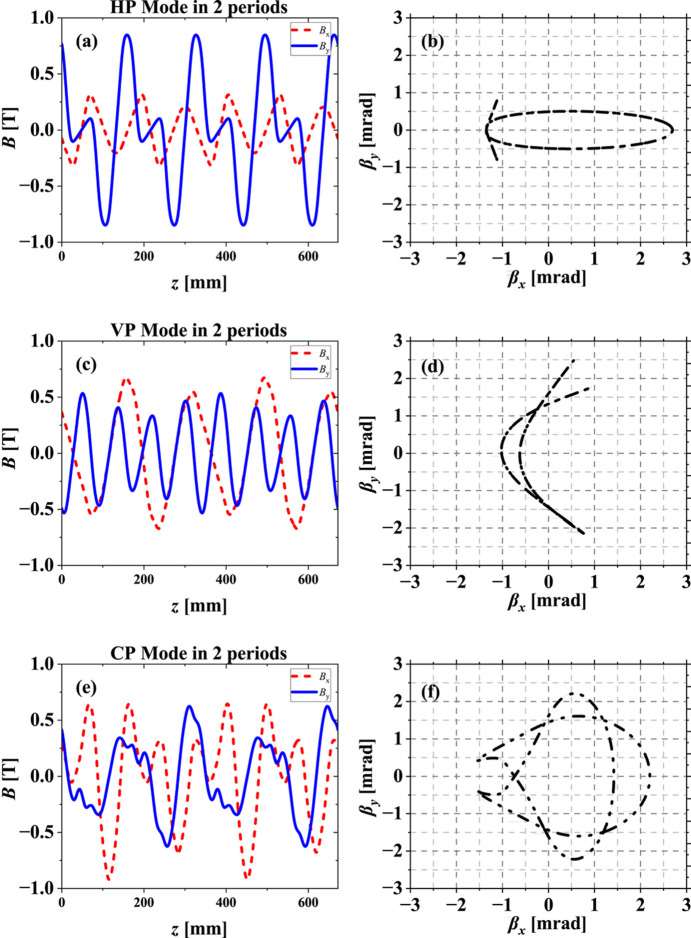
Magnetic fields (*a*, *c*, *e*) for three polarization modes in two periods of the novel AK undulator, with the corresponding velocity maps (*b*, *d*, *f*) under an electron beam energy of 2.2 GeV, respectively.

The magnetic field distribution at different polarization modes of the novel AK undulator are shown in Fig. 5 ▸. At zero phase shift of the horizontal polarization (HP) mode, the dominant field (*B*_*y*_) contributed from KNOT arrays is not a continuous sinusoidal wave, while the additional field (*B*_*x*_) determined by APPLE arrays behaves as a continuous sinusoidal wave. In vertical polarization (VP) mode and circular polarization (CP) mode, the phase shift is not zero; the field is coupled from APPLE and KNOT arrays. In any case, we can still observe the period ratio reversing from 2:3 to 3:2 between strong and weak fields, which is in contrast to the traditional AK undulator. The velocity maps are also important to explain radiation properties, as presented in Fig. 5 ▸. An obvious velocity deviation from the axis can be observed, which indicates an effective suppression for on-axis heat load. The corresponding phase shifts of different polarization modes are listed in Table 2 ▸. At these three polarization modes, to keep the same resonant photon energy, the undulator gap is set to 17 mm for HP mode and CP mode, and 20 mm for VP mode.

## 4. Radiation performance

**Figure 6:**
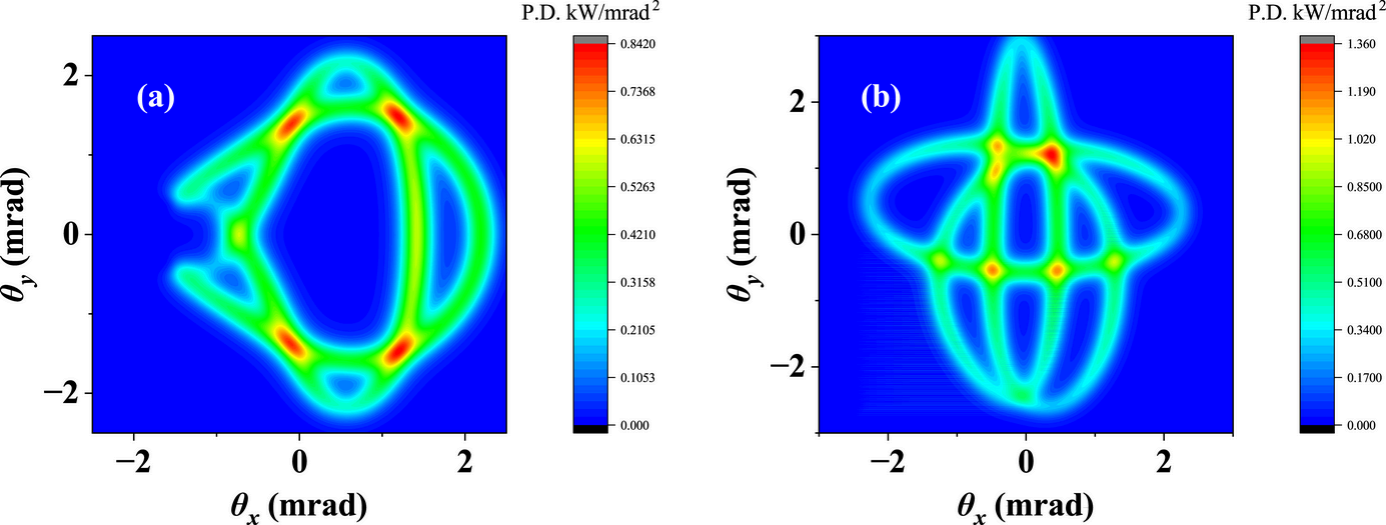
Power density distribution in CP mode at the minimum gap for the novel AK undulator (*a*) and traditional AK undulator (*b*).

The radiation performance is analysed using *SPECTRA*, with the magnetic field generated by *RADIA* (Elleaume *et al.*, 1997 ▸; Chubar *et al.*, 1998 ▸) based on the electron parameters of HALF, as listed in Table 1 ▸. With the target minimum photon energy of 6 eV and the specific undulator parameters listed above, the magnetic fields of the CP modes for the two versions of the AK undulator are given as 
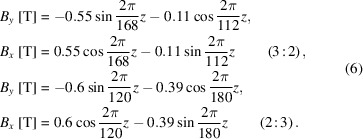
The period ratios of the APPLE to KNOT arrays are always 2:3 in these two AK undulators. However, the period ratios between the strong field and weak field in these two undulators become 3:2 and 2:3, respectively. The lengths of the periodic unit of these two undulators are set by a small difference. In CP mode of the novel AK undulator, both the array 1 and array 3 should shift 33.6 mm in the same direction, which corresponds to 3/10 of one period unit. The traditional AK undulator has a similar phase movement in CP mode. The power density distributions of CP mode for these two undulators at minimum gap are shown in Fig. 6 ▸. A good on-axis heat-load suppression effect can be observed in the novel AK undulator. The results clearly indicate that the novel AK undulator has less on-axis heat load than the traditional AK undulator.

**Figure 7:**
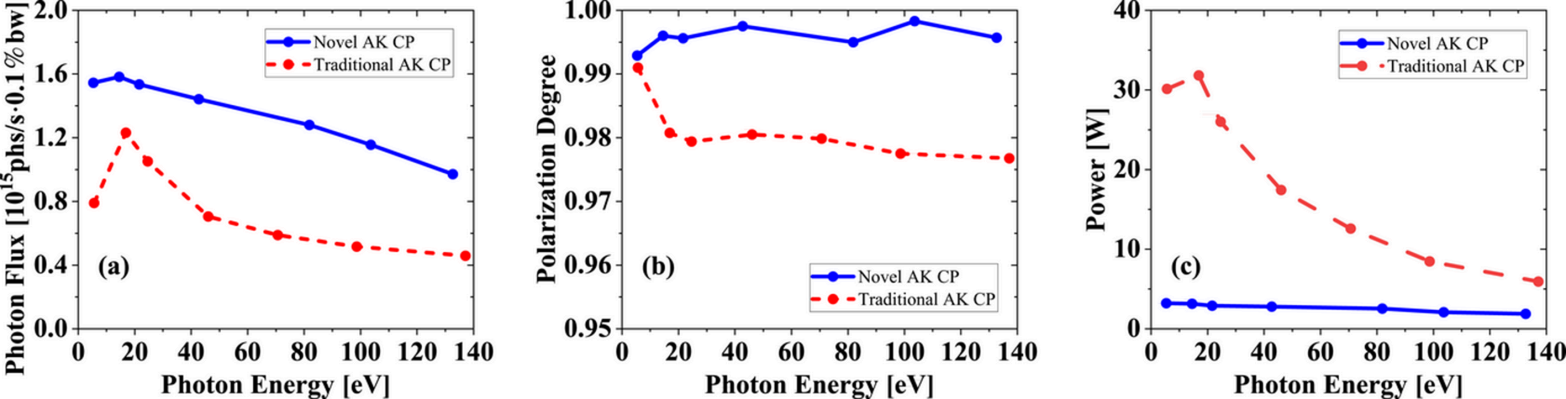
Photon flux (*a*), polarization degree (*b*), and on-axis power (*c*) in CP mode of two types of AK undulators. The acceptance angle at different photon energy always keeps two multiples of divergence.

We have systematically compared the radiation performance of CP mode between these two undulators, as shown in Fig. 7 ▸. The acceptance angle always keeps two multiples of divergence for different photon energy. An obvious improvement on flux and polarization degree can be obtained in the novel AK undulator. The flux in CP mode of the novel AK undulator is about twice that of the traditional AK undulator, with more than 1% higher polarization degree from 40 eV to 137 eV. The circular polarization degree remains above 99% and averages around 99.5% at different photon energy. The on-axis heat load of the novel AK undulator is limited to 4 W, which is about 0.15% of the total power. Although these two undulators have a small difference both in period length and period number, we can still conclude that such a great improvement in flux is mainly down to the advantage of this novel AK design. It is also highly consistent with the theoretical results. The CP mode of the novel AK undulator is closer to an APPLE-II undulator than the traditional AK undulator.

**Figure 8:**
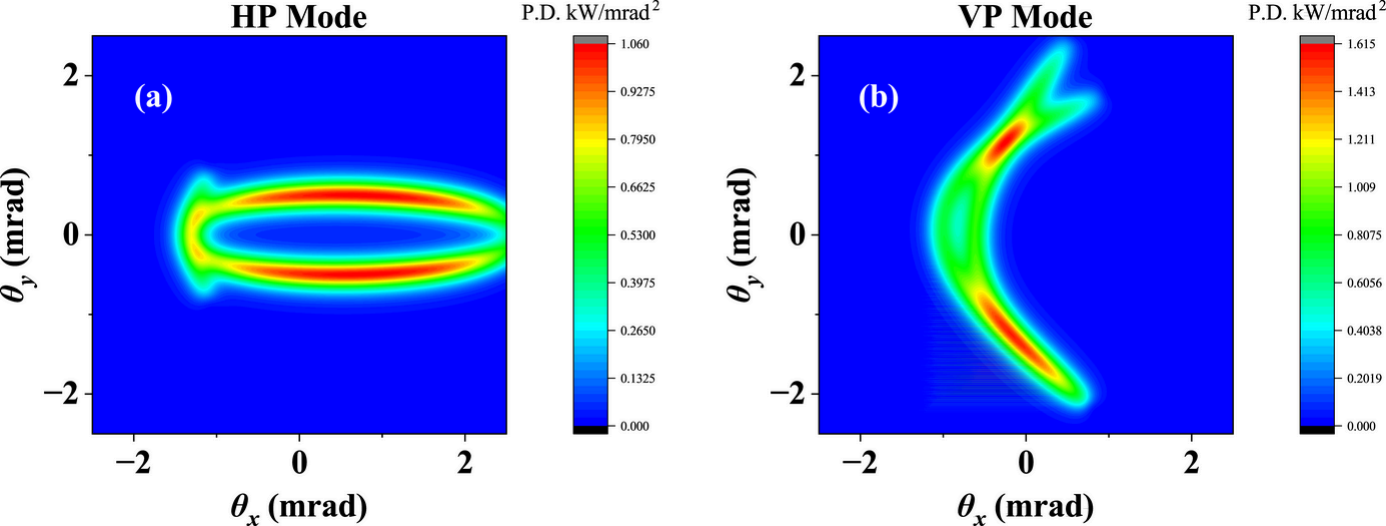
Power density distribution for HP (*a*) and VP (*b*) modes of the novel AK undulator.

**Figure 9:**
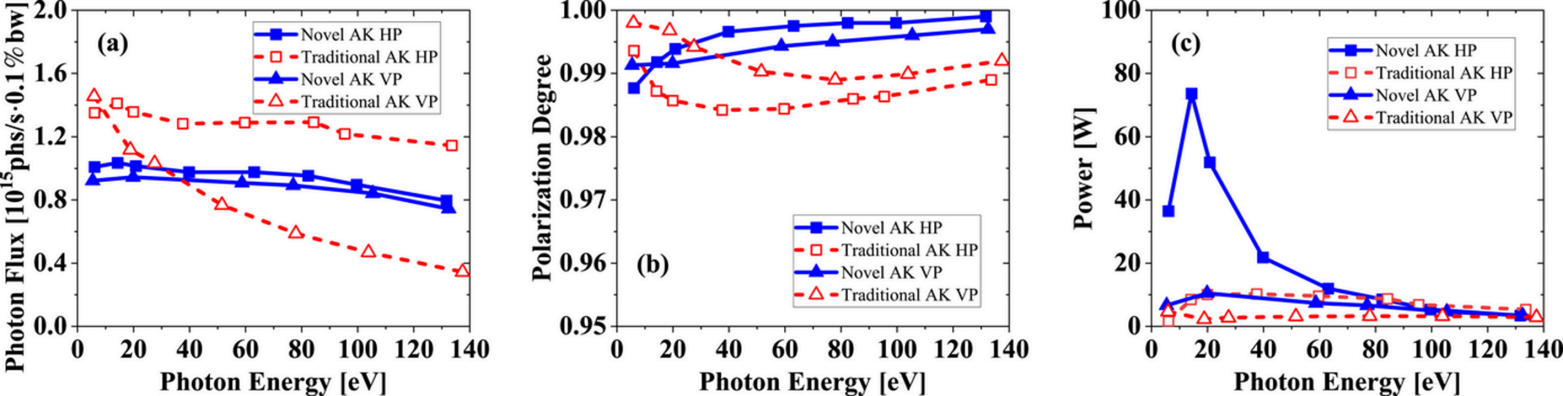
Photon flux (*a*), polarization degree (*b*), and on-axis power (*c*) in linear polarization mode. The acceptance angle at different photon energy always keeps two multiples of divergence.

A basic function of the AK undulator is generating HP radiation and VP radiation while keeping a low on-axis heat load. The proposed novel AK undulator also keeps this function. At zero phase shift, it works in HP mode. If array 1 is shifted by 3/4 of one period unit along the positive longitudinal direction and array 3 is shifted 3/4 of one period unit along the negative direction, it will work in VP mode. Fig. 8 ▸ shows the power density distribution for the HP and VP modes of the novel AK undulator at 6 eV. It still shows a good capability to suppress the on-axis heat load in linear polarization mode. The radiation characteristics of the novel AK undulator in comparison with the traditional AK undulator are shown in Fig. 9 ▸. The traditional AK undulator has a higher flux in HP mode and a lower flux in VP mode than the novel AK undulator for most of the covered photon energies. The flux difference between HP and VP modes of the novel AK undulator is small. Compared with the traditional AK undulator, it has a smooth variation in photon flux depending on photon energy, which behaves close to that of the APPLE-II undulator. For the linear polarization degree, the novel AK undulator is always above 99%, which is higher than for the traditional AK undulator. However, the additional field of the novel AK undulator is comparatively small in HP mode – its on-axis heat load is a little higher than the traditional AK undulator. Within a radius of two multiples of divergence, the maximum on-axis power reaches 73 W at a photon energy of 14 eV, which is about 8.7% of the total power. In any case, the radiation performance does not compromise significantly.

**Figure 10:**
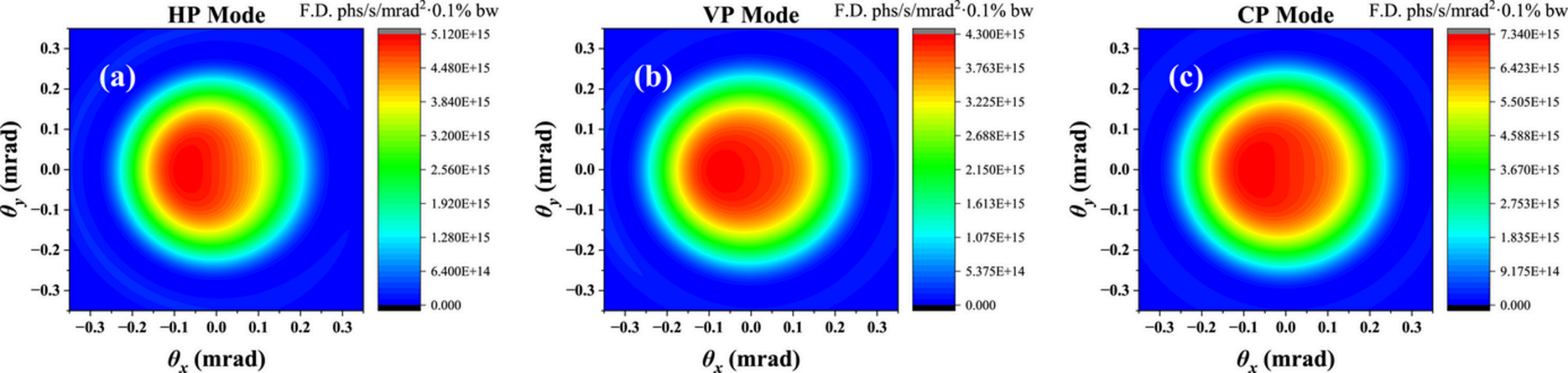
The angular flux density distribution for HP mode (*a*), VP mode (*b*) and CP mode (*c*) under the minimum working gap conditions.

Besides the total flux, polarization degree and on-axis heat load, the experiment users also care about the angular flux density distribution. In Fig. 10 ▸, we show the transverse angular distribution for different polarization modes. All three polarization modes can operate at approximately 6 eV, which can fully satisfy our experiment users on minimum photon energy. Most of the useful flux is limited to within a radius of 0.25 mrad.

## 5. Discussion and summary

**Figure 11:**
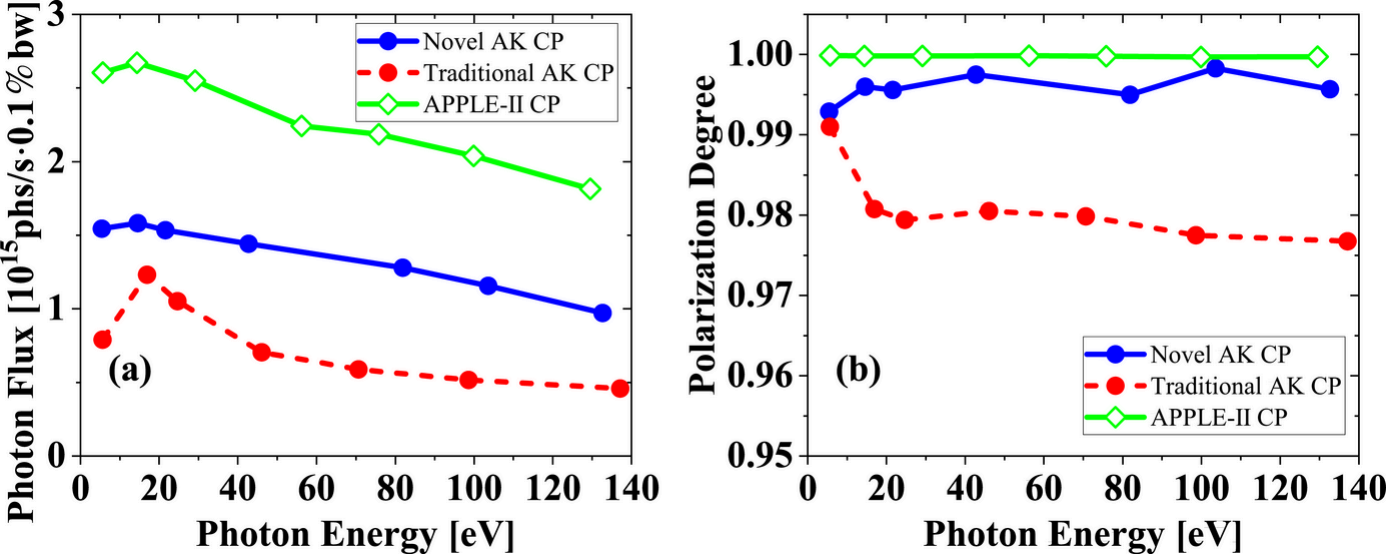
Photon flux (*a*) and polarization degree (*b*) of two versions of the AK undulator and APPLE-II undulator in CP mode. The acceptance angle at different photon energy always keeps two multiples of divergence.

This paper proposes a novel AK undulator in which the KNOT magnet array configuration is instead utilized to generate the dominant field. To reduce the contribution from additional fields, we set a small tilt magnetization angle for the merged magnet block. The APPLE and KNOT blocks are merged to form two new blocks with significantly different specifications. Both the theoretical analysis and numerical simulation indicate that selecting the KNOT field as the dominant field can effectively improve the radiation characteristics of circular polarization mode, while the radiation performance of linear polarization mode does not suffer a serious compromise. To further demonstrate the key factor causing such an obvious difference between these two AK undulators, an APPLE-II undulator is taken as a reference. The period length of the APPLE-II undulator is set to 120 mm, with a transverse block size of 40 mm × 40 mm and the same horizontal gap of 6.5 mm and same minimum gap of 17 mm. The minimum resonant photon energy reaches 5.7 eV, which is close to these two AK undulators. Fig. 11 ▸ shows the radiation performance of the CP mode for different types of undulators. The APPLE-II undulator achieves the highest flux and polarization degree, maintains a polarization degree above 99.9% across the tested photon energy range, and outperforms AK undulators. It also indicates that the additional field will deteriorate the radiation performance, which is consistent with the above results.

Obviously, the traditional APPLE-II undulator cannot fully satisfy the user demands, especially for reducing the on-axis heat load. For the AK undulator design, an optimal balance among photon flux, on-axis heat load, and polarization degree across different polarization modes requires careful optimization in the period ratio and field strength ratio. The previous developed AK undulators are also successful for suppressing the on-axis heat load in linear polarization mode. However, the radiation performance of circular polarization mode is still important to various adjustable polarization undulators. It deserves to be further considered with the other polarization modes. The proposed AK undulator aims to provide experiment users with expanded options and a deep understanding of the radiation characteristics inherent to AK type undulators.

## Figures and Tables

**Table 1:** Design parameters of the electron beam in the middle of a long straight section of HALF

Beam energy (GeV)	2.2
Average current (mA)	350
Horizontal emittance (pm rad)	176.4
Vertical emittance (pm rad)	13.2
Horizontal/vertical beta function (m)	6.834/2.536
Horizontal dispersion function (m)	0.0005

**Table 2:** Design parameters of the novel AK undulator

Complete period length 3λ_u_ (mm)	336
KNOT array (strong field) period length 1.5λ_u_ (mm)	168
APPLE array (weak field) period length λ_u_ (mm)	112
Period number	13
Dimensions of merged block (horizontal × vertical × longitudinal) (mm)	50 × 50 × 14
Dimensions of block corresponding to blank segment (horizontal × vertical × longitudinal) (mm)	50 × 5 × 14
Field strength ratio	5:1
Remanent magnetization of NdFeB (T)	1.26
Array-1/array-3 shift in horizontal mode @ 6 eV (mm)	0/0
Array-1/array-3 shift in vertical mode @ 6 eV (mm)	84 (3λ_u_/4)/−84 (−3λ_u_/4)
Array-1/array-3 shift in circular mode @ 6 eV (mm)	33.6 (3λ_u_/10)/33.6 (3λ_u_/10)

**Table 3:** Design parameters of the traditional AK undulator

Complete period length 3λ_u_ (mm)	360
KNOT array (weak field) period length 1.5λ_u_ (mm)	180
APPLE array (strong field) period length λ_u_ (mm)	120
Period number	12
Dimensions of merged block (horizontal × vertical × longitudinal) (mm)	50 × 50 × 15
Dimensions of block corresponding to blank segment (horizontal × vertical × longitudinal) (mm)	50 × 35 × 15
Field strength ratio	20:13
Remanent magnetization of NdFeB (T)	1.26
Array-1/array-3 shift ain horizontal mode @ 6 eV (mm)	0/0
Array-1/array-3 shift in vertical mode @ 6 eV (mm)	67.5 (9λ_u_/16)/−67.5 (−9λ_u_/16)
Array-1/array-3 shift in circular mode @ 6 eV (mm)	36 (3λ_u_/10)/36 (3λ_u_/10)
